# Blocking extracellular glycine uptake mediated by GlyT1 mitigates protoporphyria

**DOI:** 10.1172/JCI197344

**Published:** 2025-09-16

**Authors:** Marc Liesa

**Affiliations:** 1 Institut de Biologia Molecular de Barcelona, IBMB, CSIC, Barcelona, Catalonia, Spain.; 2 CIBERDEM, Instituto de Salud Carlos III, Madrid, Spain.

## Abstract

Accumulation of the light-reactive heme precursor protoporphyrin IX (PPIX) in blood causes protoporphyria, a disease characterized by severe pain resulting from sunlight exposure, as well as by the occurrence of liver failure in some patients. Thus, decreasing PPIX biosynthesis is a promising strategy to treat protoporphyria. In this issue of the *JCI*, Ducamp et al. report that inhibition of the glycine plasma membrane transporter GLYT1 using bitopertin decreased PPIX accumulation and ameliorated liver disease using human in vitro and mouse in vivo models. Their findings support the ongoing development of bitopertin to treat protoporphyria, while concurrently pointing to underexplored roles of glycine in erythroid cells.

## Two genetic disorders driven by protoporphyrin IX accumulation

**Figure 1:**
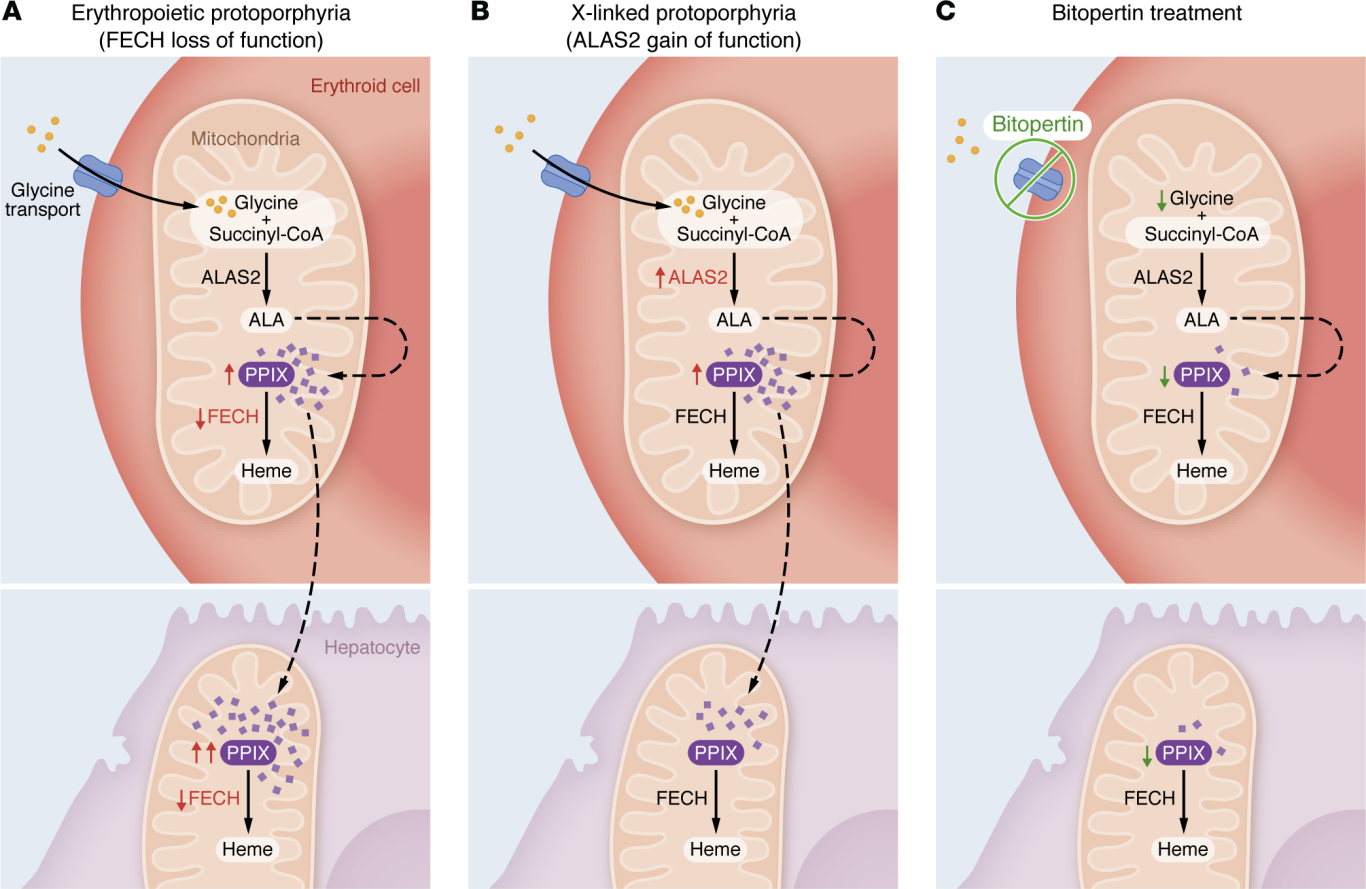
EPP and XLPP induce PPIX accumulation by different mechanisms, but blocking glycine import with bitopertin can decrease PPIX accumulation in both forms. ALAS2 catalyzes glycine and succinyl-CoA into ALA in the first reaction of heme biosynthesis in erythroid cells, but not in hepatocytes. In a downstream reaction that occurs in both erythroid cells and hepatocytes, the enzyme FECH converts PPIX into heme. (**A**) Diminished FECH function in EPP is expected to induce a larger increase in PPIX in hepatocytes due to primary PPIX accumulation in both erythroid cells and hepatocytes. (**B**) However, in XLPP, ALAS2 gain of function leads to PPIX accumulation in erythroid cells only. (**C**) Illustration shows how bitopertin-mediated blockade of the glycine transporter limits glycine availability, preventing PPIX accumulation in both conditions. Notably, although limiting glycine availability and decreasing FECH in erythroid cells was predicted to be accompanied by decreased heme biosynthesis if PPIX were to decrease, this was not observed. This points to other mechanisms by which glycine contributes to heme homeostasis and by which erythroid cells can preserve heme content independently of FECH activity.

Two genetic disorders affecting hematopoiesis converge on the pathogenic mechanism that protoporphyrin IX (PPIX), a light-reactive precursor to heme, accumulates in erythrocytes and plasma tissues. Erythropoietic protoporphyria (EPP) is caused by inactivating mutations in ferrochelatase (*FECH*), the enzyme that catalyzes the addition of iron to PPIX, the final step in heme biosynthesis (1). Thus, in EPP, decreased consumption causes PPIX to accumulate in all cell types, given that FECH expression is not restricted to erythroid cells. On the other hand, X-linked protoporphyria (XLPP) is caused by a mutation that increases the activity of amino-levulinic acid synthase 2 (ALAS2), an erythroid cell–specific enzyme that catalyzes the synthesis of δ-aminolevulinic acid (ALA), the first and rate-limiting step in PPIX and heme biosynthesis (2) (Figure 1). This means that PPIX accumulation in XLPP results from elevated PPIX production exclusively in erythrocytes. After bone marrow, the liver synthesizes the second highest amount of heme per day. However, hepatocytes depend on ALAS1 to synthesize heme instead of ALAS2. Consequently, the liver disease observed in patients with XLPP cannot be the result of a primary alteration of heme biosynthesis in the liver.

In this regard, the exact mechanisms by which erythroid-specific defects in heme biosynthesis induce liver damage are not completely understood. The predominant view is that the chronic supply of PPIX released by intact EPP-affected erythrocytes and by splenic and liver macrophages after turning over EPP-affected erythroid cells is the triggering factor that leads to acute cholestatic liver failure. Another possibility is that reactive PPIX derivatives generated by sunlight exposure cause the lysis of erythrocytes, which releases a large amount of these reactive and toxic derivatives into the serum that are then detoxified by the liver. Considering these theories, it is plausible to expect that the development and severity of liver disease will be greater in EPP, in which PPIX accumulates in both erythrocytes and hepatocytes, compared with XLPP, in which PPIX accumulation is restricted to erythrocytes. The hepatocytes of patients with EPP are exposed to primary PPIX accumulation in their cytosol as well as PPIX and its derivatives imported from the serum (Figure 1). The current study by Ducamp et al. reveals evidence from mouse models in support of this expectation, as well as a therapeutic avenue with potential to ameliorate liver disease in EPP and PPIX (3).

## How bitopertin revealed glycine’s role in heme biosynthesis

GLYT1 is a plasma membrane glycine transporter that is highly expressed in neurons (4), but is also present in bone marrow and induced by erythroid differentiation (5). Bitopertin was developed as a GLYT1 inhibitor that blocks glycine reuptake in glia. Its original purpose was to improve symptoms of schizophrenia by increasing glycine in the synaptic cleft to activate NMDA receptors. However, bitopertin treatment for schizophrenia was discontinued in phase III of the study (6). Safety data obtained in the schizophrenia studies showed that bitopertin decreased hemoglobin synthesis to nonconcerning levels in healthy humans (baseline −0.03 to −1.07 g/dL) (7). ALAS2 transforms glycine and succinyl-CoA into ALA; thus, bitopertin’s reduction of hemoglobin levels could be due to its effect on this glycine-dependent step of PPIX synthesis. The fact that GLYT1 is also expressed in the bone marrow is consistent with glycine being essential to synthesize PPIX and heme.

With this precedent of bitopertin decreasing hemoglobin in healthy humans, Ducamp et al. set out to determine whether bitopertin could prevent PPIX accumulation and liver disease in mouse models of EPP and XLPP (8). Moreover, Ducamp et al. studied the actions of bitopertin in a human-derived erythroid cell line engineered to harbor EPP-causing *FECH* mutations (K562-EPP cells), in EPP patient–derived erythroblasts, and in erythroblasts derived from healthy donors with *FECH* knocked down (3).

## Bitopertin ameliorates protoporphyria and liver damage in mice

In this context, bitopertin decreased both ALA and PPIX content in human K562-EPP cells (3) (Figure 1). However, bitopertin was unable to decrease heme content in these same cells (3). This unexpected result raises the question of how bitopertin-treated K562-EPP cells can harbor the same amount of heme as untreated counterparts, despite the simultaneous inhibition of glycine uptake and impaired FECH activity. Bitopertin treatment may induce plasticity mechanisms in these erythrocytes to preserve heme content in response to further reductions in heme synthesis. One plausible mechanism could be upregulation of processes that enable extracellular heme uptake. Another could be downregulation of heme oxygenases or other systems that degrade excess heme.

In the mouse models of EPP and XLPP, bitopertin treatment at optimized doses reduced the accumulation of PPIX in erythrocytes and plasma. Whereas untreated XLPP mice rarely developed liver disease, liver damage was present in untreated EPP mice. The observation that bitopertin prevented liver damage in some EPP mice and that this effect was maintained throughout treatment provides a strong rationale for investigating the clinical use of bitopertin in patients with EPP as well as those with XLPP.

## Examining the efficacy of bitopertin across stages of erythroid maturation

Additional data in the study showed that bitopertin did not decrease PPIX content in erythroid precursors isolated from the bone marrow of protoporphyric mice. Rather, bitopertin’s largest effect of decreasing PPIX content was observed in reticulocytes — immature, enucleated erythrocytes that will soon become circulating RBCs. As the glycine consumption necessary to produce ALA occurs inside the mitochondria, the highest demand for glycine imported by GLYT1 is expected to occur when mitochondrial mass, and thus heme synthesis, peaks. During erythroid differentiation, mitochondrial mass peaks in polychromatophilic erythroblasts in the bone marrow (8, 9). Next, it is widely thought that enucleation is triggered when hemoglobin content reaches some threshold level, and then the process of elimination of mitochondria by mitophagy starts in parallel to enucleation in orthochromatic erythroblasts. Thus, it appears that bitopertin efficacy is lowest at the erythrocyte differentiation stage, coinciding with the peak in mitochondrial mass, function, and heme synthesis. However, it is also known that the few mitochondria remaining in reticulocytes can still produce heme.

These findings generate very interesting questions: Do polychromatophilic erythroblasts exclusively use endogenously synthesized glycine to produce heme, enabled by their high mitochondrial mass? Or can polychromatophilic erythroblasts activate mechanisms of extracellular heme import (10) when the competition for glycine to support maximal globin and heme synthesis limits glycine availability? Is heme biosynthesis in reticulocytes more dependent on extracellular glycine uptake because enucleation and mitophagy abrogate the capacity to synthesize glycine? Is glycine needed for other processes key for erythrocyte differentiation, such as oxidative stress protection mediated by glutathione (11, 12)?

## Conclusions and considerations

In all, the study by Ducamp et al. shows bitopertin’s efficacy in decreasing PPIX content in erythroblasts from humans and in reticulocytes from mice with protoporphyria. In addition, bitopertin decreased the occurrence of liver damage in the mouse model of EPP. Therefore, this study might inspire new research on heme homeostasis during erythroid differentiation and shows the promise of bitopertin treatment in preventing the symptoms of protoporphyria.

## Version 1 09/16/2025

Electronic publication
